# ASSOCIATION BETWEEN ACANTHOSIS NIGRICANS AND OTHER CARDIOMETABOLIC RISK FACTORS IN CHILDREN AND ADOLESCENTS WITH OVERWEIGHT AND OBESITY

**DOI:** 10.1590/1984-0462/;2018;36;3;00017

**Published:** 2018

**Authors:** Heloísa Marcelina da Cunha Palhares, Paula Cunha Zaidan, Fernanda Cristina Mattos Dib, Adriana Paula da Silva, Daniela Cristina Silva Resende, Maria de Fátima Borges

**Affiliations:** a Universidade Federal do Triângulo Mineiro, Uberaba, MG, Brasil.

**Keywords:** Acanthosis nigricans, Pediatric obesity, Insulin resistance, Risk factors, Acantose *nigricans*, Obesidade pediátrica, Resistência à insulina, Fatores de risco

## Abstract

**Objective:**

To evaluate the presence or absence of acanthosis nigricans and its association with metabolic alterations in a group of obese and overweight children and adolescents.

**Methods:**

A cross sectional study of 161 overweight children and adolescents, who were divided into two groups, according to presence or absence of acanthosis nigricans. Anthropometric measurements (body mass index, skinfolds, abdominal circumference), blood pressure, laboratory tests (fasting glycemia, insulin, lipid profile, triglycerides, uric acid, transaminases) and homeostasis model assessment index.

**Results:**

The acanthosis nigricans group represented 51.5% of the sample. The mean age was similar between groups. The group with acanthosis nigricans presented higher body mass index, Z score of body mass index, body fat percentage, abdominal circumference (p<0.0001), systolic (p=0.006) and diastolic blood pressure (p=0.002). There was no significant difference in the analysis of lipid profile, except for the high-density cholesterol, which was lower (p=0.003) in the group with acanthosis. On the other hand, uric acid (p<0.0001), fasting glycemia (p=0.006), insulin (p<0.0001), glutamic oxalacetic transaminase (p<0.0001), and homeostasis model assessment index (p<0.0001) were significantly higher in the group with acanthosis nigricans.

**Conclusions:**

Acanthosis nigricans in overweight and obese children and adolescents is associated with elevation of body fat, blood pressure, insulin and homeostasis model assessment index, indicating that it is a clinical marker associated with the metabolic syndrome.

## RESUMO

**Objetivo:**

Avaliar em um grupo de crianças e adolescentes com obesidade e sobrepeso a presença ou não de acantose *nigricans* e sua associação com alterações metabólicas.

**Métodos:**

Estudo transversal envolvendo 161 indivíduos com excesso de peso, que foram divididos em dois grupos, segundo a presença ou não de acantose *nigricans*, e nos quais foram obtidas medidas antropométricas (índice de massa corporal, pregas cutâneas, circunferência abdominal), pressão arterial, análises laboratoriais (glicemia de jejum, insulina, perfil lipídico, triglicerídeos, ácido úrico, transaminases) e o índice *homeostasis model assessment*.

**Resultados:**

O grupo com acantose *nigricans* representou 51,5% da amostra. A média de idade foi semelhante entre os grupos. O grupo com acantose *nigricans* apresentou maiores índice de massa corporal, escore Z do índice de massa corporal, percentual de gordura corporal, circunferência abdominal (p<0,0001) e pressão arterial sistólica (p=0,006) e diastólica (p=0,002). Não houve diferença significativa na análise do perfil lipídico, exceto o colesterol de alta densidade, que foi menor (p=0,003) no grupo com acantose. Já o ácido úrico (p<0,0001), a glicemia de jejum (p=0,006), a insulina (p<0,0001), a transaminase glutâmica oxalacética (p<0,0001) e o índice *homeostasis model assessment* (p<0,0001) foram significativamente maiores no grupo com acantose *nigricans*.

**Conclusões:**

Acantose *nigricans* em crianças e adolescentes com sobrepeso e obesidade esteve associada à elevação dos índices de adiposidade corporal, pressão arterial, insulina e *homeostasis model assessment*, indicando-a como marcador clínico associado à síndrome metabólica.

## INTRODUCTION

Childhood obesity is a chronic disease with multifactor etiology which is becoming increasingly prevalent.^1^ In, Brazil, the prevalence of childhood obesity is 14.1% - a percentage based on data from a meta-analysis encompassing January 2008 to May 2014^2^ -, and according to the World Obesity Federation, from 2009 to 2011, the prevalence of overweight and childhood obesity was 26.7% among males and 34.6% among females.^3^ This high prevalence of childhood obesity is associated with increased metabolic changes considered to be risk factors for cardiovascular diseases and diabetes mellitus type 2.^4^

Metabolic syndrome is defined as a group of disorders that includes, in addition to obesity, insulin resistance **-** playing a central role in its development **-** dyslipidemia, arterial hypertension and other metabolic disorders associated with cardiovascular disease.^5^ Individual factors that make up metabolic syndrome pose cardiovascular risks, and the syndrome is nothing but the sum of these. Although the classification of metabolic syndrome is controversial, cardiometabolic risk factors are known to exist since pediatric age.^5^ ^,^ ^6^

The prevalence of insulin resistance in childhood is not well known yet^7^ and its diagnosis is very difficult due to the lack of a single method capable of estimating the level of individual sensitivity to insulin.^8^ Regarding insulin resistance, the gold standard has been hyperinsulinemic-euglycemic clamp,^8^ but this is a complex test, especially when it comes to its execution in the pediatric age group. Other indicators such as HOMA-IR (homeostasis model assessment ***-*** insulin resistance) and oral glucose tolerance test have been used. The questioning is that hyperglycemia rarely occurs in childhood and, therefore, the evaluation of insulin levels is consensual to diagnose insulin resistance, but data point out the need to establish reference curves for a proper assessment.^9^

Other clinical signs of insulin resistance that patients may present, in addition to obesity, are increased waist circumference and acanthosis nigricans (AN), a very common finding often associated with hyperinsulinemia and childhood obesity. Apparently, the only AN predictive factors are hyperinsulinism and severe obesity, not age or pubertal stage.^10^ However, the literature lacks studies conducted with children and adolescents. The area mostly affected by AN in children is the neck (93-99%), followed by the axilla (73%).^11^ This study evaluated a group of children and adolescents with obesity and overweight as to the presence or absence of AN and its association with metabolic alterations.

## METHOD

This was a cross-sectional study conducted with 1,125 children and adolescents (aged 5 to 19 years old) from public and private schools in the city of Uberaba, Minas Gerais. After anthropometric evaluation, 364 children and adolescents were classified as overweight and referred for evaluation at the pediatric endocrinology clinic of *Universidade Federal do Triângulo Mineiro* (UFTM), from February 2013 to July 2014. In total 172 individuals participated and, after exclusion by chronic diseases (osteogenesis imperfecta ***-*** 1 individual) or not agreed participation (10 individuals), the final sample was constituted of 161 children and adolescents with overweight and obesity.

Patients were divided into two groups: group 1 (G1), with 83 children and adolescents with AN, and group 2 (G2), with 78 children and adolescents without AN.

Weight was measured on a platform-type digital electronic scale with capacity of up to 150 kg and 100-g precision (Filizola^®^, São Paulo, Brazil). The subjects were wearing light clothing, barefoot, positioned vertically in the center of the scale, and standing still. Height was measured in upright position, feet united in parallel, barefoot, on a wall stadiometer, model E150A, graduated up to 220 cm and divided in millimeters (Tonelli^®^, Criciúma, Brazil). The height measurement was made three times in a row, with calculation of average to obtain the final result. Body mass index (BMI) was calculated dividing weight (kg) by squared height (m^2^) (kg/m^2^). Body mass index (Z-BMI) score was used to define nutritional status of individuals,^12^ aided by the WHO-Anthro Plus 2007 software (World Health Organization, Geneva, Switzerland). Individuals were categorized as overweight (+1 ≤ Z-BMI <+2) and obese (Z-BMI ≥ +2).

Physical examination consisted of a detailed evaluation of the skin in search of clinical signs of insulin resistance, which is evidenced by the presence of AN, visually assessed in the neck, axilla, elbows, and inguinal region.

The abdominal circumference (AC) was measured with the individual in upright position, at the midpoint between the lower margin of the last rib and the upper border of the iliac crest, after a normal expiration, with inextensible metric tape in millimeters.

Skin folds were measured with a scientific plicometer (Cescorf^®^, Porto Alegre, Brazil) with 0.1mm sensitivity, 85mm reading range and ± 10 g/mm^2^ pressure.^13^ The bicipital, tricipital, suprailiac and subscapular skin folds were measured. Body fat percentage (%) was calculated based on tricipital and subscapular skinfolds sum as a criterion for equation choice and calculated on Slaughter equations.^14^

Blood pressure (BP) was measured in the right arm, positioned at the height of the heart with the hand palm facing upwards, while the individual was sitting and resting. A stethoscope, a sphygmomanometer and cuffs appropriate for the age group and arm circumference were used. Three measurements were taken with a minimum interval of two minutes and mean values were calculated and classified according to the US *National High Blood Pressure Education Program Working Groupon High Blood Pressure in Children and Adolescents* ^15^, recommended by the I Guidelines for the Prevention of Atherosclerosis in Childhood and Adolescence, by the Brazilian Society of Cardiology.^16^ According to these criteria, blood pressure levels ≥95 percentile (P95) for age, gender and height were considered high.

Pubertal staging was classified according to the presence of secondary sexual features in both genders, as per the criteria proposed by Marshall and Tanner.^17^ ^,^ ^18^ As for laboratory tests, after 10 to 12 hours of fasting, a blood sample was collected by peripheral venous puncture for the following tests: glycemia, insulin, total cholesterol, HDL cholesterol, LDL cholesterol, triglycerides, glutamic oxalacetic transaminase (GOT), glutamate-pyruvate transaminase (GPT), Gamma-glutamyltransferase (gamma GT) and uric acid.

Serum concentrations of total cholesterol, HDL cholesterol, triglycerides, uric acid and gamma GT were measured by the enzymatic colorimetric method^19^, and glycemia by the enzymatic method with hexokinase.^19^ To determine TGO and TGP hepatic enzymes, the enzymatic method was used^19^ and all samples were processed in COBAS 6000 module C501 (Roche Diagnóstica, São Paulo, Brazil). Serum LDL cholesterol concentrations were calculated on the Friedewald equation.^20^

Fasting insulin was determined by the electrochemiluminescence method and processed in the COBAS 6000 module C601. Two comparative analyzes were performed to evaluate hyperinsulinemia: the cutoff point by the I Atherosclerosis Prevention Guidelines in Childhood,^16^ considering it altered when greater than 15 mIU/mL; and the criteria by Almeida et al.,^9^ considering altered the mean of insulin values described adjusted by gender and age group, added to two standard deviations.

 HOMA-IR index was used to evaluate insulin resistance after being obtained by the calculation of the product of insulin (microU/mL) and fasting glycemia (mmol/L) divided by 22.5.^21^ The cutoff used was higher or equal to 2.5 for prepubertals^22^ and greater or equal to 3.43 for pubertals of both genders.^23^

In statistical analysis, individuals were divided into two groups, according to the presence (G1) or absence (G2) of AN. Data were submitted to descriptive analysis based on absolute and percentage frequencies, and measures of centrality and dispersion. The Kolmogorov-Smirnov test with Liliefors correction was used to verify normality of variables, while the homogeneity of variances between groups was checked by the Levene test.

In comparisons between groups, the Student-t test was used when data had normal variance distribution and homogeneity, and the Mann-Whitney test when these conditions were not met. For comparison or association between categorical variables, the chi-square test was used.

According to their distribution, Pearson’s or Spearman’s correlation coefficients were used to investigate the association between variables as per groups analyzed.

The level of significance for all inferential procedures was set at 5%. The software STATISTICA, Statsoft, version 10, was used in statistical procedures (StatSoft South America, São Caetano do Sul, Brazil).

The project was approved by the Human Research Ethics Committee of UFTM, protocol 2479. The children, adolescents and their parents/caregivers were informed about the research project and signed the informed consent form.

## RESULTS

G1 (with AN, n=83) was composed of 27 males and 56 females, representing 51.5% of the total sample, and G2 (without AN, n=78) represented 48.4% of the total sample. with 31 males and 47 females. Mean age was similar between groups (p=0.70); gender and pubertal stage (p=0.34 and p=0.50, respectively) were also not statistically significant in comparison. These clinical and laboratory data are listed in Table 1.

**Table 1:** Clinical and laboratory characterization of groups with and without acanthosis.

	Presence of acanthosis (n=83)	Absence of acanthosis (n=78)	p-value
Age (years)^a^	11.7±2.9	10.8±3.1	0.07
Gender (male/female)^b^	27/56	31/47	0.34
Pubertal stage (pre-pubertal/pubertal)^b^	31/52	33/45	0.5
BMI (kg/m²)^a^	27.4±3.4	23.4±3.6	<0.0001
Z-BMI^c^	2.5 (1.2-4.9)	1.88 (1.0-3.9)	<0.0001
Body fat %^a^	43.0±8.9	36.1±9.0	<0.0001
AC (cm)^a^	84.2±9.9	75.0±10.4	<0.0001
SBP (mmHg)^a^	109.8±10.1	105.6±9.4	0.006
DBP (mmHg)^a^	71.4±8.1	67.7±6.9	0.002
Total cholesterol (mg/dL)^c^	164.3 (88.7-230.5)	158.9 (103.5-279.4)	0.87
HDL-cholesterol (mg/dL)^a^	43.0±10.7	48.1±10.8	0.003
LDL-cholesterol (mg/dL)^a^	102.8±28.4	99.4±32.5	0.48
Triglycerides (mg/dL)^c^	86.0 (41.0-286.0)	83.0 (31.0-445.0)	0.28
TG/HDL-c^c^	2.2 (0.7-9.8)	1.82 (0.6-10.3)	0.054
Fasting glycemia (mg/dL)^a^	89.2±10.5	84.1±12.5	0.006
Insulin (microUI/mL)^c^	15.1 (4.6-117.2)	11.3 (1.4-35.7)	<0.0001
HOMA-IR^c^	3.2 (0.8-28.7)	2.3 (0.3-7.1)	<0.0001
GOT (U/L)^a^	19.7±5.9	18.9±5.9	0.41
PGT (U/L)^c^	11.5 (1.1-72.0)	11.3 (3.1-35.2)	<0.0001
Gamma GT (U/L)^c^	19.3 (0.2-55.9)	14.8 (5.7-38.0)	<0.0001
Uric acid (mg/dL)^a^	4.9±1.0	4.2±1.0	<0.0001

BMI: body mass index; Z-BMI: body mass index score; AC: abdominal circumference; SBP: systolic blood pressure; DBP: diastolic blood pressure; TG/HDL-c: relation triglycerides/HDL-cholesterol; HOMA-IR: homeostasis model assessment - insulin resistance; GOT: glutamic oxalacetic transaminase; PGT: pyruvic glutamic transaminase; Gamma GT: Gamma-glutamyltransferase; ^a^Students’ *t* test: values expressed in mean ± standard deviation; ^b^chi-square test; ^c^Mann-Whitney test: values expressed median (V_min._-V_max._).

Statistical analysis of anthropometric data showed a significant difference between G1 and G2 as to BMI (p<0.0001), Z-BMI (p<0.0001), body fat percentage (p<0.0001) and AC (p<0.0001), confirming that patients with AN had higher adiposity index (Table 1).

In analysis of mean blood pressure values, a statistically significant difference was found between groups for both systolic (SBP, p=0.006) and diastolic blood pressure (DBP, p=0.002), with higher blood pressure levels in G1. However, upon classification of pressure levels above P95, there was no difference in the frequency of pressure change between groups (SBP, p=0.18 and DBP, p=0.21). As for percentage, G1 had 10.8% of individuals with SBP above P95 and 12.1% with DBP above P95, while the group without AN, had 5.2 and 6.4% of values above P95, respectively.

Regarding laboratory data, groups were not statistically different when it came to lipid profile, except for HDL cholesterol, with lower mean in G1 compared to G2 (p=0.003) (Table 1).

Although no child was diagnosed with type 2 diabetes mellitus in this study, fasting glycemia and insulin were significantly higher in G1 when compared to G2 (p=0.006 and p<0.0001, respectively). Similarly, the HOMA-IR index was higher in G1 (p<0.0001) (Table 1), as it was altered in 54.2% of participants in this group and in 32% of G2 members. Basal insulin concentrations ranged from 4.63 to 117.2 microUI/mL in G1 and from 3.1 to 35.2 microUI/mL in G2; they were above 15 microUI/mL in 50.60% of children and adolescents of G1 and in 21.8% of G2 members. Based on the criteria by Almeida et al.,^9^ the frequency of altered levels of insulin was 66.2% in the group with AN and 50% in the group without AN.

Serum concentrations of uric acid (p<0.0001) and GPT (p<0.0001) were significantly higher in G1 (Table 1).

Correlations between clinical and laboratorial data of G1 and G2 are shown in Tables 2 and 3. There was a significant positive association of BMI and AC with triglycerides in G1 only (Table 2). The analysis of correlation of insulin and HOMA-IR with blood pressure and triglycerides was also significant in G1 only, as well as the negative correlation with HDL cholesterol (Table 3).

**Table 2:** Simple linear correlation between body mass index and abdominal circumference with clinical and laboratory parameters in the groups of overweight and obese children and adolescents with and without acanthosis.

	BMI				AC			
	With AN		Without AN		With AN		Without AN	
	r	p-value	r	p-value	r	p-value	r	p-value
BMI^a^	-	-	-	-	0.84	0.00	0.89	0.00
Z-BMI^b^	0.60	0.00	0.55	0.00	0.50	0.00	0.49	0.00
Body fat %^a^	0.67	0.00	0.63	0.00	0.69	0.00	0.63	0.00
AC^a^	0.84	0.00	0.89	0.00	-	-	-	-
SBP^a^	0.42	0.00	0.53	0.00	0.47	0.00	0.52	0.00
DBP^a^	0.36	0.00	0.41	0.00	0.35	0.00	0.43	0.00
Total cholesterol^b^	0.20	0.06	0.01	0.92	0.32	0.00	-0.03	0.74
HDL-cholesterol^l^	-0.11	0.29	-0.02	0.82	-0.15	0.18	-0.08	0.49
LDL-cholesterol^l^	0.16	0.14	0.04	0.72	0.30	0.00	0.03	0.78
Triglycerides	0.25	0.02	0.10	0.34	0.28	0.01	0.14	0.21
Fasting glycemia^a^	0.63	0.05	0.11	0.33	0.08	0.43	0.12	0.29
Insulin^b^	0.49	0.00	0.46	0.00	0.47	0.00	0.44	0.00
HOMA-IR^b^	0.47	0.00	0.47	0.00	0.48	0.00	0.45	0.00
GOT^a^	0.02	0.82	-0.38	0.00	0.04	0.66	-0.37	0.00
PGT^b^	0.26	0.01	0.02	0.82	0.25	0.02	0.25	0.02
Gamma GT^b^	0.25	0.02	0.18	0.14	0.38	0.00	0.19	0.12
Uric acid^a^	0.40	0.00	0.46	0.00	0.45	0.00	0.51	0.00

BMI: body mass index; AC: abdominal circumference; AN: acanthosis nigricans; r: linear correlation coefficient; Z-BMI: body mass index score; SBP: systolic blood pressure; DBP: diastolic blood pressure; HOMA-IR: homeostasis model assessment - insulin resistance; GOT: glutamic oxalacetic transaminase; PGT: pyruvic glutamic transaminase; Gamma GT: Gamma-glutamyltransferase; ^a^Pearson’s linear correlation; ^b^Spearman’s linear correlation.

**Table 3:** Simple linear correlation between resistance index Homeostasis model assessment and insulin with clinical and laboratory parameters in the groups of overweight and obese children and adolescents with and without acanthosis.

	HOMA-IR*				Insulin*			
	With AN		Without AN		With AN		Without AN	
	r	p-value	r	p-value	r	p-value	r	p-value
BMI	0.47	0.00	0.47	0.00	0.49	0.00	0.46	0.00
Z-BMI	0.22	0.00	0.28	0.01	0.22	0.04	0.21	0.06
Body fat %	0.35	0.00	0.41	0.00	0.37	0.00	0.47	0.00
AC	0.47	0.00	0.45	0.00	0.47	0.00	0.44	0.00
SBP	0.32	0.00	0.12	0.26	0.36	0.00	0.15	0.16
DBP	0.24	0.02	-0.04	0.71	0.33	0.00	0.02	0.08
Total cholesterol	0.16	0.14	0.08	0.48	0.11	0.31	-0.01	0.89
HDL-cholesterol	-0.25	0.01	-0.08	0.47	-0.22	0.04	-0.12	0.26
LDL-cholesterol	0.15	0.16	0.08	0.45	0.10	0.37	-0.01	0.88
Triglycerides	0.35	0.00	0.12	0.26	0.36	0.00	0.15	0.16
Fasting glycemia	0.29	0.00	0.31	0.00	0.08	0.44	0.06	0.58
Insulin^b^	0.96	0.00	0.94	0.00	-	-	-	-
HOMA-IR	-	-	-	-	0.96	0.00	0.94	0.00
GOT	-0.18	0.10	-0.19	0.09	-0.24	0.02	-0.34	0.00
TGP	0.12	0.27	0.12	0.29	0.06	0.59	-0.03	0.80
Gama GT	0.32	0.00	0.04	0.72	0.29	0.00	0.05	0.66
Uric acid	0.28	0.00	0.18	0.13	0.23	0.03	0.19	0.11

HOMA-IR: homeostasis model assessment - insulin resistance; AN: acantose *nigricans*; r: linear correlation coefficient; BMI: body mass index; Z-BMI: body mass index score; AC: abdominal circumference; SBP: systolic blood pressure; DBP: diastolic blood pressure; GOT: glutamic oxalacetic transaminase; PGT: pyruvic glutamic transaminase; Gama GT: Gamma-glutamyltransferase; *Spearman’s linear correlation.

## DISCUSSION

In this study, we evaluated changes that would be associated with the presence of AN in children and adolescents with obesity and overweight. The group with AN (G1) had higher BMI, Z-BMI, AC, body fat percentage, BP, insulin, HOMA IR, TGO, gamma GT and uric acid values, but lower HDL cholesterol values. In addition, there was a correlation between HOMA-IR, insulin and blood pressure levels. The ERICA^24^ study reported a 2.6% prevalence of metabolic syndrome among Brazilian adolescents aged 12 to 17 years, and the frequency of its components, in decreasing order, was: high AC, low HDL cholesterol, BP, triglycerides and glucose.^24^ In a study conducted with prepubertal children with obesity or overweight aged 2 to 11 years old, Madeira et al.^22^ reported 27.9% of the sample with AN, 61.4% with increased AC, 55.7% with low HDL cholesterol, and 16.4% of subjects meeting the criteria for metabolic syndrome diagnosis. In our study, 51.5% of the patients with AN and a tendency to more significant changes in blood pressure levels, AC and laboratory tests, indicating that AN is associated with metabolic syndrome in overweight and obese children and adolescents.

We also found a statistically significant difference as to fasting glycemia (higher in the group with AN), HDL cholesterol (lower in the group with AN) and mean SBP and DBP (higher in the group with AN), although there was no significant difference between groups in relation to blood pressure levels ≥ P95.

In addition, Klucznik et al.^11^ assessed 194 children and adolescents between the ages of 2 and 18 years and showed an association between AN and BMI, AC, insulin and HOMA-IR. These variables were significantly higher in the AN group. The authors also reported AN associated with non-white skin color, and that this ethnicity presented 5.4 times more chances of developing AN. On the other hand, Guran et al.^10^, after evaluating 160 children with obesity, detected 67 (41.8%) with AN, but reported similar fasting glycemia, total cholesterol, HDL cholesterol, LDL cholesterol, SBP and DBP between groups with and without AN. However, they found significantly higher insulin and HOMA-IR values in the AN group. Important to note that Juonala et al.,^25^ after evaluating 6,328 individuals, showed that those who maintained overweight from childhood to adulthood had higher relative risk of hypertension. Thus, the association between acanthosis and higher blood pressure levels indicates that this clinical sign should lead to the suspicion and early detection of cardiovascular alterations, also implying early treatment in the pediatric age group.

Insulin resistance is defined as a state of subnormal biological response to serum levels of this hormone and leads to hyperinsulinism as an attempt to reach adequate physiological response.^7^ The increase in adipose tissue in childhood is the most probable initial event leading to changes in glucose metabolism and insulin resistance triggering, so clinical and laboratorial indicators of insulin resistance in obese children must be sought after.^24^ When analyzing insulin resistance, using a cut-off of 15 mIU/L for altered insulin, Guran et al.^10^ found 54.71% of the sample meeting criteria for insulin resistance in the group with AN and only 17.8% in the group without AN.

On the same cutoff, we found 50.6% of altered insulin in the group with AN and 21.8% in the group without AN. However, according to Almeida et al.^9^, analysis of insulinemia per age and gender was higher in both groups, with and without AN (66 and 50%, respectively), reinforcing the hypothesis that a fixed cutoff point for insulinemia leads to underdiagnosis of hyperinsulinism, as insulin does not behave evenly throughout the entire childhood, puberty and young adulthood.

The Bogalusa Heart Study reported that individuals with persistently high insulin levels had higher BMI, blood pressure, total cholesterol, LDL cholesterol, triglycerides and glycemia values, and lower HDL cholesterol compared to individuals with lower insulin levels. In addition, persistently high insulin was associated with a more proneness to developing hypertension, dyslipidemia and obesity in young adults.^26^ Other investigations have also demonstrated the association between hyperinsulinemia in childhood and progression to type 2 diabetes mellitus in young adults,^27^ ^,^ ^28^ which reinforces the need for early intervention.

There was a correlation between HOMA-IR and insulin with anthropometric variables and triglycerides levels. Similar data were also found by other studies with children and adolescents,^8^ ^,^ ^29^ which described a relevant positive association between BMI and insulin, HOMA-IR and BMI, HOMA-IR and AC, HOMA-IR and triglycerides, confirming the findings of our study and showing that insulin resistance is associated with changes in risk for metabolic disorders at maturity. However, it should be emphasized that, although fasting insulin dosage and HOMA-IR are relevant in epidemiological studies to check for insulin resistance, there is no justification for this screening in clinical practice when assessing overweight children.^7^ In this group, the search for clinical metabolic changes such as AN is highly relevant and represents the need for prevention strategies.

Uric acid has a relevant positive correlation with BMI, AC and insulin levels. Miranda et al.^30^ correlated increased serum uric acid levels with insulin resistance and found that increase of 1 mg/dL in serum uric acid levels was responsible for a 91% increase in chance of developing insulin resistance. Although there the literature is not consensual as to reference values of this marker in children and adolescents, uric acid dosage appears to be a good predictor of cardiometabolic risk in young age groups.^30^

Our study has limitations, mainly because of its transversal design, so temporal and causal relations cannot be established. Despite this, one can conclude that AN is a clinical finding that is strongly associated with metabolic alterations, insulin resistance, hyperinsulinemia, obesity and metabolic syndrome. Thus, identifying it is important so that early screening, research and intervention can occur, enabling the prevention of associated diseases, improving quality of life and reducing comorbidities related to obesity.
